# Personals

**Published:** 1918-11

**Authors:** 


### Personals.

Dr. Oscar Davis, Anderson, has been appointed Assistant State Health Officer, to fill the unexpired term of Dr. A. L. Lincecum, who resigned to enter the Army medical service.

Dr. S. S. Robinson, formerly of New Orleans, is now located at El Campo, Texas, where he has bought property and expects to make a home.

Complaint has been filed against Dr. H. P. Whatley of Fort Worth and Mineral Wells in the United States Commissioner's Court, charging violation of the narcotic law. He waived examing trial and furnished $1,500 bond.

Dr. W. F. Hasskarl, Brenham, has been elected Health Officer of Washington County.

Dr. H. F. Vermillion, El Paso, will be Superintendent of the Southern Baptist Sanatorium, a new institution. The building will be erected in the near future.

Dr. John M. Holt, Surgeon, U. S. Public Health Service, has been appointed City Health Officer of Houston for the period of the war.

Dr. John M. Neel has been appointed medical member of the

South Dallas board to succeed Dr. T. C. Gilbert, now an officer in the Medical Corps of the army.

Dr. R. B. Vales of Wharton, Texas, has reecntly moved to Hot Springs, Ark.

Dr. D. H. Clark of Utopia has accepted a position with his cousin, Dr. O. E. Clark, of Schulenberg.

Dr. T. T. Jackson, San Antonio, has received his majority. His address is Major T. T. Jackson, Camp Pike, Little Rock, Arkansas, Base Hospital.

